# A Painless Patient Is a Pleased Patient

**Published:** 1904-05

**Authors:** 


### A PAINLESS PATIENT IS A PLEASED PATIENT.

There's not much comfort in a name. Your patient doesn't care whether it's a cavity, an exposed nerve, or an ulcerated tooth. If there's pain, that's enough for the patient. If it's toothache, face-ache, or headache, no matter! It is nerve-ache; there's pain. Believe it! Pain is often all there is to it. Stop the pain and you please the patient. Morphine? If you like! But why create "a habit," check digestion, retard absorption, diminish renal secretion, constipate,, and reduce the flow of bile? Antikamnia & Codeine Tablets stop pain, reduce fever. That's the whole story. No disturbance of stomach, bowels, kidneys, liver or heart. Dentists when operating should administer one Antikamnia & Codeine tablet every hour giving one shortly before beginning the operation. One Antikamnia & Codeine tablet before and one after extracting a tooth, will prevent the severé pain and consequent nervousness. Toothache, facial neuralgia, and earache can be greatly relieved with one Antikamnia & Codeine tablet every hour or two.

